# An integrative model of perseverative thinking

**DOI:** 10.1080/19585969.2025.2481658

**Published:** 2025-05-23

**Authors:** Lorenzo Mattioni, Ana V. Nikčević, Francesca Ferri, Marcantonio M. Spada, Carlo Sestieri

**Affiliations:** a Department of Neuroscience, Imaging and Clinical Sciences – and ITAB, Institute for Advanced Biomedical Technologies, G. d’Annunzio University of Chieti-Pescara, Italy; b Department of Psychology, Kingston University, Kingston Upon Thames, UK; c School of Applied Sciences, London South Bank University, London, UK

**Keywords:** perseverative thinking, self-regulation, executive functions, allostasis, memory reconsolidation

## Abstract

People spend most of their waking hours detached from external stimuli, remembering the past, foreseeing the future, imagining situations in which they did not attend or that have never existed, or, simply, thinking. Such a process is crucial for mental health. A common feature of many mental disorders is recurrent stress-related thoughts, the so-called ‘perseverative thinking’. In this review, we describe how perseverative thinking represents a dysfunctional self-regulatory strategy that maintains and increases the effects of mental suffering and arises from the maladaptive interplay between discrepancy monitoring, strategy selection, executive regulation, and information representation. We further argue that perseverative thinking can change how the mind represents the world through memory updating, resulting in an increased perceived need for regulation of the external and internal inputs. Lastly, we propose a new integrated model incorporating the different features of perseverative thinking, offering a more unified perspective on psychopathology.

## Introduction

Worrying to prepare for an anxiety-inducing situation or ruminating to analyse a negative past event and prevent its recurrence serves to allocate cognitive resources in advance for self-regulatory purposes. These processes can occur spontaneously, but they can also be intervened on. In this view, they are automatic, similar to the act of walking, which can occur without active thought but can also be intentionally directed, but different from other involuntary processes, like a heartbeat, over which we lack direct control. This review focuses on conceptualising this kind of repeated mental activity, which we would call ‘perseverative thinking’.

Repetitive cognitions can be adaptive or maladaptive, depending on their function. For instance, excessive worry may cause anxiety, whereas non-perseverative problem-solving (e.g. concrete, focused thinking on a specific problem) can lead to more adaptive outcomes. Persistent rumination often worsens mood, as opposed to self-reflection, which is goal-directed and constructive, facilitating psychological adjustment. In this view, perseverative thinking is not just a repetition of knowledge but indicates a continuous representation of salient information, often triggered by external events, e.g. a stressful situation, or internal states, e.g. negative emotions, which can continue despite changing circumstances. Its valence can be positive, as in desire thinking, which revolves around rewarding goals, or negative, as in worry and rumination, which focus on threats or losses. Furthermore, perseverative thinking can be self-oriented (e.g. self-critical rumination) or other-oriented (e.g. anger rumination) and may focus on the past, present, or future. For example, worry tends to be future-oriented, while rumination reflects on past events (see BOX1 for examples and distinctions of perseverative thinking).

Mental disorders, despite their apparent diversity, share a common feature: they involve something that people do with their minds. Generally, emotional distress is short-lived because people can flexibly manage negative thoughts and beliefs. However, ineffective cognitive self-regulation plans can arise and continuously hinder the natural reduction of mental suffering (Beck 1979; Ellis 2010). In this review, we propose that perseverative thinking has a key role in this maladaptive outcome. While perseverative thinking may temporarily reduce emotional arousal by allowing disengagement from aversive input, it simultaneously inhibits emotional processing and reinforces maladaptive cognitive patterns with long-term consequences (Foa and Kozak 1986). For instance, worrying can prolong and intensify anxiety by maintaining attentional biases towards perceived threats, ultimately preventing the natural dissipation of negative emotional states and their consequences over time (Borkovec et al. 1998). Thus, while the purpose of this process is to analyse and derive insights about the event or state in an attempt to resolve or alleviate mental suffering, it is typically counterproductive.

A study that used a smartphone app to gather 250,000 data points on subjects’ thoughts, feelings, and actions as they went about their lives shows that people spend half of their waking hours thinking about something other than what they are doing, and this typically makes them unhappy (Killingsworth and Gilbert 2010). While different disorders, such as substance abuse or post-traumatic stress disorder, imply repetitive mental representations of very different events, and their related context, the brain mechanisms that allow us to repeatedly represent those events in our minds may not be so different (Taylor et al. 1998). Event recollection, imagination, and prediction are thought to depend on similar cognitive processes (Schacter et al. 2007), supported by common neural mechanisms (Mullally and Maguire 2014), and associated with both effective and ineffective self-regulatory processes (Taylor et al. 1998). A wide range of mental disorders appears to involve similar chains of hierarchical top-down cognitive control strategies that engage perseverative thinking to cope with distressing thoughts and negative emotional experiences (Ehring and Watkins 2008; Aldao and Nolen-Hoeksema 2010).

‘The map is not the territory’ is a metaphor used in general semantics to illustrate the distance between something and its description. However, these two levels are often confused in clinical research: people’s symptoms (i.e. the territory) are represented through a map of symptom patterns which are then considered as ‘true’ manifestations of mental disorders. Whilst this classification system may distinguish between different disorders based on the co-occurring symptoms, in the real world, it is extremely rare to find someone who manifests psychological distress according to a single nosographic description. The heterogeneous nature of the psychopathological categories is particularly problematic due to the overlap of symptoms across categories that are treated differently (Allsopp et al. 2019). These considerations underscore the need for a more nuanced approach to understanding mental health, one that moves beyond rigid diagnostic boundaries.

Research on the dimensionality and comorbidity of mental disorders suggests that they are, in fact, manifestations of relatively few underlying core dimensions (Krueger and Eaton 2015). Removing the distinctions between diagnoses opens new ways of classifying mental health problems and suggests alternative conceptualizations and treatments (Dalgleish et al. 2020). Accordingly, suggestions have been made that the focus should be on common psychopathological processes that underly many different mental disorders, both in their conceptualisation and treatment (Sauer-Zavala et al. 2017). This paradigm shift allows for a more unified understanding of mental health, highlighting the cognitive processes that may contribute to the development and maintenance of diverse disorders.

Box 1. Examples of perseverative mental activities characterising mental disorders versus non-perseverative mental activities.

*Examples of perseverative thinking:*

- *Worry is* an attempt at mental problem-solving concerning situations with a potentially negative outcome. It is primarily future-oriented, though it can sometimes involve concerns about present or past events with future implications (Meyer et al. 1990; Borkovec et al. 1998). It is commonly seen in anxiety disorders, such as generalised anxiety disorder and social anxiety disorder.
- *Brooding* is a subtype of rumination consisting of a response to negative emotions and characterised by a passive focus on one’s symptoms, their causes, and their consequences (Nolen-Hoeksema et al. 2008; Aldao et al. 2010). It is strongly associated with depression and contributes to the maintenance and exacerbation of negative mood states.
- *Self-critical rumination* is a repetitive cognitive activity involving judgmental, condemning, and attacking thoughts towards the self. Self-critical rumination is distinct from self-criticism given its persistent and cyclical nature, often involving prolonged reflection on perceived personal failures or shortcomings. It is commonly observed in individuals with low self-esteem and those suffering with social anxiety. It is often associated with feelings of shame and guilt (Gilbert et al. 2004; Smart et al. 2016; Milia et al. 2021).
- *Anger rumination* involves repetitive and persistent reflection on experiences that trigger this emotion, including memories of events, thoughts and feelings, and plans for revenge (Sukhodolsky et al. 2001; Denson et al. 2012; Quan et al. 2021). It is thought to perpetuate feelings of anger and is linked to heightened aggression.
- *Perseverative thinking focusing on food, body shape, and weight* is common across eating disorders, such as anorexia nervosa and bulimia nervosa, where such thoughts contribute to disordered eating behaviours and body image disturbances (Shafran et al. 2004; Park et al. 2011; Sala et al. 2019).
- *Repetitive analytical thinking focused on intrusive thoughts* is a hallmark of obsessive-compulsive disorder, where individuals repeatedly analyse intrusive thoughts. It involves excessive worry about the potential consequences of these thoughts, leading to repeated mental problem-solving, reassurance-seeking, or mental checking. These processes are aimed at neutralising perceived threats or preventing feared outcomes but, paradoxically, they end up reinforcing the cycle of intrusive thoughts and compulsive mental activity (Rachman 1998; Cohen and Calamari 2004; Wahl et al. 2019).
- *Desire thinking* represents a voluntary cognitive process aimed at prefiguring desired target images, information, and positive memories to regulate craving, an important index for addiction disorders such as substance use disorders and behavioural addictions (Kavanagh et al. 2004; Caselli and Spada 2015; Allen et al. 2017)

*Examples of non-perseverative thinking*:

- *Problem-solving* is not considered perseverative thinking as experimental studies have found that repetitive self-focus characterised by more concrete, experiential processing has more beneficial outcomes than repetitive self–focus characterised by abstract, evaluative processing (Watkins and Moulds 2005);
- *Mind wandering*, the shift of attention away from events in the external environment towards self-generated thoughts and feelings (Smallwood and Schooler 2015), is not considered repetitive thinking when not associated with cognitive inflexibility, autonomic rigidity, and mood worsening (Ottaviani et al. 2013);
- *Self-Reflection* is the deliberate and constructive process of examining one’s experiences, thoughts, and feelings. Unlike brooding, self-reflection is motivated by curiosity or epistemic interest in the self and is associated with openness to experiences, without the negative focused cyclical nature of repetitive negative thinking. It can be conceptualised as an adaptive process of problem-solving or self-regulation, which can serve to increase self-knowledge and facilitate psychological adjustment (Trapnell and Campbell 1999; Treynor et al. 2003; Takano and Tanno 2009);
- *Intrusive thoughts, e.g. obsessions* (Wahl et al. 2011) *or trauma-related memories* (Michael et al. 2007), are not perseverative thinking since they are not an evaluation aimed at self-regulation. However, intrusive thoughts can become the object of perseverative thinking if one starts worrying or ruminating about them;
- *Delusions,* i.e. fixed beliefs that are not amenable to change in light of conflicting evidence, are not perseverative thinking but rather represent a disturbance in prediction-errors updating of beliefs about the world resulting from an abnormal neurochemical pattern (Fletcher and Frith 2009) that differs from the one concerning perseverative thinking.

## Views on perseverative thinking

The literature offers different paradigms that account for perseverative thinking. We present an overview of some of the most influential theories of this mental activity.

*Control Theory* (Carver and Scheier 1982), grounded in a cybernetic framework, posits that biased self-regulatory behaviour stems from suboptimal negative feedback loops. These loops involve monitoring discrepancies between the current and a desired reference state, activating corrective measures to attain this reference point, and, crucially for perseverative thinking, discontinuing these measures upon reaching a subjectively satisfactory outcome (Miller et al. 1960). The process of developing functional self-regulated mental behaviour depends on the internalisation, evaluation, and maintenance/revision of these standards (Berzonsky 1997). In this view, the long-term discomfort resulting from perseverative thinking is dependent on the quality and quantity of mental resources devoted to these feedback loops, rather than the specific content of the thoughts (Ruscio et al. 2001).

In the psychotherapeutic field, *the Self-Regulatory Executive Function model* (Wells and Matthews 1996; Wells 2019) was developed to explain perseverative thinking by dividing cognitive processes into three hierarchically interconnected levels, operating at different timescales. The lowest level represents a network of elementary processing units, activated sequentially by specific inputs (Norman and Shallice 1986). These stimulus-response patterns can become automatic, continuously processing information without influences from the higher levels. The second level constitutes genuine perseverative thinking and represents a controlled process of redundant evaluation and regulatory feedback loops (Fergus et al. 2012; Nordahl and Wells 2019). Similarly to the mechanisms described in the previous paragraph, these loops are sustained by persistent monitoring of discrepancies. The highest level regards self-knowledge and metacognitive beliefs involving declarative characteristics, i.e. conscious beliefs about the nature and usefulness of thoughts, and procedural characteristics, i.e. automatic cognitive strategies and habits driven by these beliefs which sustain and reinforce the inflexible monitoring processes maintained by the lower levels (Spada et al. 2008). This negative and perseverative thinking style is represented within the Cognitive Attentional Syndrome (CAS) (Wells and Matthews 1996) consisting of worry/rumination, threat monitoring, and unhelpful coping strategies.

For the *Impaired Disengagement Hypothesis* (Koster et al. 2011), stressors interfering with individuals’ goals trigger analytical and self-critical thoughts. These thoughts persist until the individual either reaches a solution or engages in emotion regulation processes, whether automatic or deliberate. Successful disengagement of attention from negative thoughts then allows to reappraise or distract from the situation altogether by focusing attention on other stimuli (De Raedt and Koster 2010). This theory posits that during this stage of conflict signalling, impaired attentional control may hinder disengagement, thereby sustaining attentional focus on maladaptive perseverative thinking (Koster et al. 2006).

The construct of *Repetitive Negative Thinking (RNT)* (Ehring and Watkins 2008) was developed within the field of clinical research to provide a transdiagnostic framework for describing perseverative thinking (McEvoy et al. 2013). RNT is a repetitive and passive process focused on negative content, which is perceived as relatively uncontrollable, unproductive, and mental capacity-capturing (Ehring et al. 2011). Notably, RNT is not maladaptive per se but can have either positive or negative consequences according to the valence of mental content, the individual disposition in a certain situation, and the level of construal, where high-level abstract construal represents the desirability and importance of outcomes and low-level concrete construal indicates the feasibility and planning of outcomes (Watkins 2008).

Embracing a physiological perspective, *the Perseverative Cognition Hypothesis* has evolved within the stress research field (Brosschot et al. 2006). The inability to effectively regulate stress adaptation, primarily due to the inadequate functioning of allostatic responders, leads to prolonged exposure to neural, endocrine, and immune stress mediators that adversely affect different systems (McEwen 1998). According to the Perseverative Cognition Hypothesis, since a stressor does not lead to prolonged activation per se, but only when it lasts over time, perseverative thinking might represent the crucial mechanism that mediates a prolonged physiological response to stressors (Brosschot et al. 2005, 2006). By continuously representing stress-related information, perseverative thinking eventually leads to various physiological negative outcomes, affecting the hemodynamic profile, the immune functioning, and the endocrine system (Ottaviani et al. 2016, 2017; Brosschot et al. 2017).

The reviewed conceptualizations of perseverative thinking differ in terms of the levels of explanation. We believe that these perspectives, although not well integrated, represent different maps of the same territory represented by perseverative thinking, which centres on the maladaptive cognitive effort aimed at managing unwanted emotions that involves visiting, over and over, the same mental representations. Our proposed integration builds upon these various theories, combining their most relevant aspects to offer what we believe is a more comprehensive understanding of perseverative thinking. Crucially, we focus on four interrelated processes that collectively explain how perseverative thinking perpetuates psychopathology across cognitive, physiological, and neural levels: executive self-regulation (allocation of cognitive resources to manage stress-related information), strategy selection (metacognitive schemas guiding coping styles), allostasis (physiological anticipation of stressors), and memory updating (reconsolidation of emotional-motivational features).

In particular, the current framework is grounded in Control Theory, which highlights how suboptimal feedback loops and inefficient allocation of mental resources contribute to the persistence of maladaptive thinking. Incorporating the SREF model, the model outlines the hierarchical structure of cognitive processes underlying perseverative thinking, from automatic stimulus-response patterns to metacognitive beliefs and self-regulation strategies that perpetuate these cycles. Drawing from the Impaired Disengagement Hypothesis, the framework emphasises how maladaptive attentional control makes it difficult to disengage from perseverative thinking, thereby prolonging the focus on salient information. Additionally, building on the RNT hypothesis, the current perspective addresses the transdiagnostic nature and clinical implications of perseverative thinking, emphasising its occurrence across diverse mental health conditions. Finally, it integrates the Perseverative Cognition Hypothesis, which links prolonged cognitive focus on stressors to adverse physiological outcomes.

We believe that the present integrative effort allows us to better differentiate between the various subcomponents of perseverative thinking, such as its role in executive self-regulation, allostasis, and memory updating. In the next sections, we will first explore the components of self-focused thinking, and then examine its relation to executive self-regulation, strategy selection, allostasis, and memory updating. We conclude with a proposal for a comprehensive neurocognitive model of perseverative thinking that differentiates between its various subcomponents and clarifies their roles in mental health. By incorporating neurobiological, cognitive, and therapeutic elements into a unified perspective, we therefore aim to offer a more comprehensive understanding of how perseverative thinking functions across different levels of explanation.

## Constraints of self-focused thinking

The ability to reconstruct past experiences or simulate future scenarios provides a great evolutionary advantage. This process relies on memory, which supplies the foundational elements for generating predictions and shaping behaviour (Suddendorf and Corballis 2007). When we remember, sparse traces of the original experiences are actively integrated into a coherent representation that simulates the past event (Schacter et al. 2012). These reconstructions also serve as building blocks for simulating hypothetical futures or alternative scenarios (Buckner and Carroll 2007; Spreng et al. 2009). However, as we will discuss in the present review, this process does not always result in an adaptive foresight, as it can become maladaptive when it fuels repetitive, inflexible patterns of thought, i.e. perseverative thinking.

Self-focused thinking, whether about the past, future, or hypotheticals, requires the engagement of executive-control resources for its sustenance (Smallwood 2013) to ensure a decoupling of the current focus of attention from immediate perceptual input (Kam and Handy 2013). This decoupling process allows transient thoughts to persist but, as a consequence, creates conflicts whit incoming perceptual information. The ability to internally represent past or future events can be both spontaneous, i.e. triggered by associative memory processes in the absence of explicit intention (Smallwood and Schooler 2006), or voluntary, e.g. during goal-directed activities (Baddeley 2012). Spontaneous instances often reflect lapses in executive control processes, where task-irrelevant thoughts intrude into awareness (McVay and Kane 2010) but also voluntarily, when motivation for external tasks wanes, and individuals metacognitively disengage to prioritise self-generated content (Seli et al. 2015).

**Figure 1.**
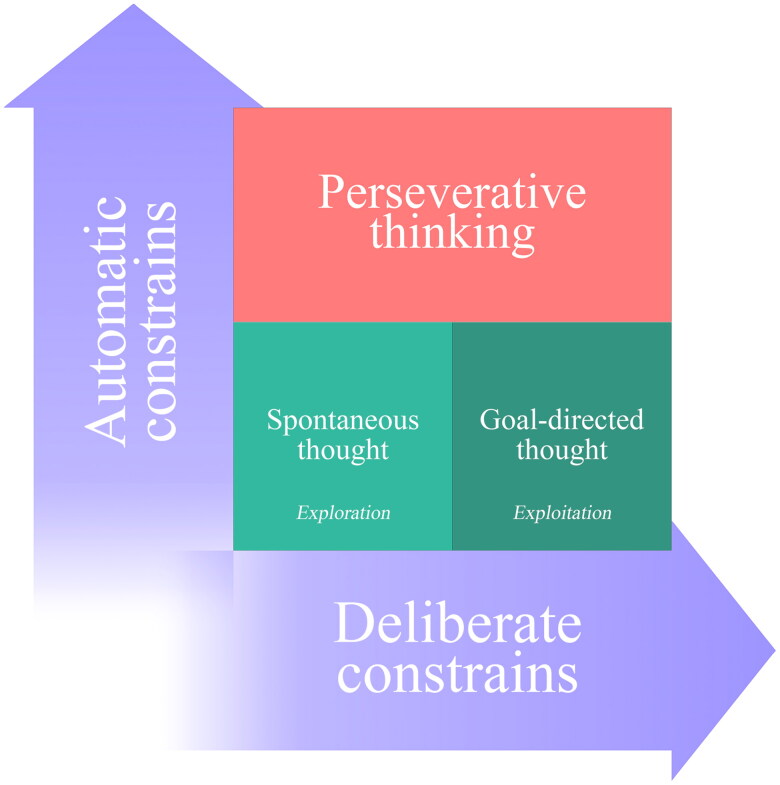
The potential variability of the present cognitive landscape depends on automatic constraints imposed on thought. Adapted from Christoff et al. (2016).

Christoff and colleagues (2016) proposed that the difference between voluntary and involuntary event representation lies in the transition between deliberate and automatic constraints. Deliberate constraints (Christoff 2012), implemented through cognitive control processes (Miller and Cohen 2001), are strongest during goal-directed thought and weaker during mind-wandering. Automatic constraints, a family of mechanisms that operate outside cognitive control to hold attention on a restricted set of salient information (Jonides and Yantis 1988; Todd et al. 2012), reduce the potential variability of mental content depending on the context (Figure 1). Perseverative thinking arises when automatic constraints dominate at their strongest levels, rigidly restricting thought to repetitive, self-relevant themes (Christoff et al. 2016). Unlike adaptive forms of mental time travel (e.g. flexible planning), perseverative thinking reflects a failure to transition between constraint levels adaptively, consequently trapping cognition in inflexible cycles of overgeneralised, self-referential processing.

Thus, the very mechanisms that enable adaptive mental time travel also form the substrate for maladaptive perseveration, highlighting how the flexibility of self-generated thought can give way to rigid, self-reinforcing loops when regulatory constraints falter.

## Executive control mechanisms in perseverative thinking

The negative consequences of perseverative thinking can be prevented by mental flexibility, a mechanism defined as the ability to switch between mental operations (Bonanno and Burton 2013). This flexibility derives from three core processes: the understanding of the context, the repertoire of regulation strategies one possesses, and the feedback monitoring ability (Armbruster et al. 2012). Flexibility emerges from the efficient combination of diverse executive function subcomponents (Diamond 2013; Dajani and Uddin 2015). Although cognitive flexibility helps protect mental health (Lydon-Staley et al. 2019), perseverative thinking is still uniquely linked to symptoms, even when accounting for psychological inflexibility (Fergus et al. 2013). Successful self-regulation is subserved by basic mechanisms of executive function, such as working memory operations, behavioural inhibition, and task-switching (Hofmann et al. 2012). Cognitive processes involved in executive functions continuously interact with those responsible for attention control, emotion regulation, and stress response (Blair and Ursache 2011) in both automatic and volitional ways (Munakata et al. 2011).

As executive functions are strongly involved in self-regulation, they are supposed to play a role in a variety of clinical phenomena. For example, the maintenance of the task goals, and the use of this information to provide top-down orienting of behavioural responses, are impaired in different psychopathologies (Friedman et al. 2008). Different deficits of executive functions can lead to the same disorder (Snyder et al. 2015). Furthermore, even if different types of perseverative thinking share common features (Brosschot et al. 2006; Ehring and Watkins 2008), they are nonetheless associated with distinct processes that support self-regulation. For example, angry rumination is associated with deficient task switching, whereas depressive rumination with a deficit in inhibiting prior mental sets (Whitmer and Banich 2007), and worry with lower general working memory capacity (Moran 2016; Gustavson et al. 2020). However, while these associations are robust when measured through self-reported executive functions in daily life, laboratory-based task performance often fails to replicate these findings (Åström et al., 2018; Mennies et al. 2021). This discrepancy may reflect the fact that questionnaires capture two distinct processes: executive functions and affect. This suggests that perseverative thinking may show more consistent associations with executive functions in the context of highly affective situations. From this perspective, the emphasis should shift from identifying which specific executive function is impaired to understanding how these impairments interact with the maladaptive patterns of neurocognitive system interactions in causing mental disorders. Psychological distress, therefore, can be conceptualised as a consequence of maladaptive control processes applied over time, rather than stemming from a general deficit in cognitive control abilities (Mattioni et al. 2023).

In summary, the relationship between perseverative thinking and executive functions highlights the intricate interplay between cognitive control processes and affective regulation. While cognitive flexibility and other executive functions are crucial protective factors for mental health, their role cannot be understood in isolation from the broader neurocognitive systems and contextual influences that shape self-regulation.

## Choosing perseverative thinking

**Figure 2.**
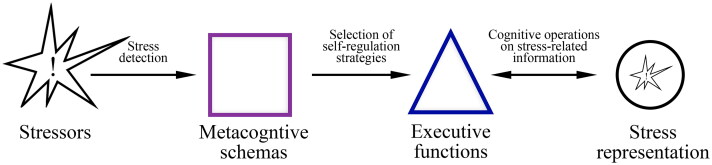
In perseverative thinking, cognitive self-regulation involves the use of executive functions to decrease the level of stress detected by continuously operating on stress-related information. This process is mediated by metacognitive schemas, which prefer this self-regulation strategy over potential alternative strategies. The arrows represent the direction of the action between the various agencies.

Early maladaptive schemas, consisting of memories, emotions, and sensations elaborated throughout the lifespan, fuel the motivation to perform perseverative thinking (Riso et al. 2006; Dozois et al. 2009; Thimm 2010). Indeed, maladaptive schemas moderate the effect of psychological flexibility on psychopathology (Fischer et al. 2016). Paradoxically, although these schemas cause suffering, they are maintained because they represent what is known and predictable, providing a sense of security and protection against uncertainty. One can also be attracted by those situations that strengthen schemas, making it difficult not only to modify them, but also to recognise their dysfunctionality (Young et al. 2006). Thus, the maintenance of early maladaptive schemas predicts perseverative thinking (Orue et al. 2014), and, in turn, such surface-level thoughts contribute to perpetuating those schemas (Calvete et al. 2013). The preference for certain coping styles associated with early maladaptive schemas is explained by an individual’s perceived metacognitive ability to regulate their stress and emotions (Ke and Barlas 2020). Metacognition refers to any knowledge or cognitive process that is involved in the appraisal, control, and monitoring of thinking (Hagen and Hjemdal 2012). The term was coined by John Flavell (1979) to indicate the general schemas and awareness of cognitive phenomena. Metacognitive schemas include knowledge about oneself, strategies, and when and why to use these strategies, while metacognitive awareness is the conscious monitoring of cognition, e.g. planning, comprehension of the situation, and evaluation of the efficacy of monitoring processes and strategies (Lai 2011). Metacognition has been recognised as an important factor in the development and maintenance of different clinical outcomes (Wells 2002), moderating the relationship between perceived stress and negative emotions (Spada et al. 2008). For example, a person can choose to worry to cope with anxiety because of implicit metacognitive beliefs stating that worry is both a useful strategy and an inevitable reaction to anxiety (Wells 2011, 1995). Notably, inflexible metacognitive schemas can develop during childhood (Esbjørn et al. 2015) as a outcome of negative experiences and anxious attachment and their maintenance explains the tendency for perseverative thinking in adulthood (Myers and Wells 2015). Importantly, various disorder-specific types of perseverative thinking are linked to metacognitive schemas (Wells 1995; Papageorgiou and Wells 2003; Gwilliam et al. 2004; Bennett and Wells 2010; Olstad et al. 2015; Spada et al. 2015). If inflexible metacognitive schemas inhibit adjustments of mental behaviour and support perseverative thinking as the only coping strategy, the subsequent discrepancy-monitoring process will never detect the desired result, i.e. the cessation of the state of distress. Consequently, distress will increase, and perseverative thinking will continue in a self-reinforcing cycle (Figure 2).

## A continuous state of anticipation

It is now well accepted that the main index of pathological stress is not the difference between the baseline activity and a specific physiological activation, but rather the total area under the curve obtained when adding the duration of the stress-related process to the equation (Juster et al. 2010). In fact, human psychopathological symptoms are better detected in sustained rather than phasic paradigms (Davis et al. 2010). Perseverative thinking is defined by a mixture of automatic and strategic processing characteristics (Beck and Clark 1997) at the service of allostasis, the autonomic anticipation of a potentially stressful event (Schulkin et al. 1994). There are several circumstances in which our organism may either be overstimulated or not perform normally due to frequent activation of systems that promote allostasis, such as the autonomic nervous system and the hypothalamus-pituitary-adrenal axis (Sterling and Eyer 1988). The inability to shut off allostatic mediators leads to the gradual shift of the homeostatic set point and thus to the gradual accumulation of allostatic load (McEwen 1998).

The increased activity of the autonomic nervous system, caused by perseverative thinking, can continue for hours after the perseveration has finished (Brosschot et al. 2007; Galbiati et al. 2018). Accordingly, subliminally presented, complex stress-related cues can influence peripheral vascular resistance (van der Ploeg et al. 2017) and, as a result, facilitate implicit memory for negative emotional material (Luethi et al. 2008). In addition, hidden sources of stress may also cause the implicit cognitive-emotional networks of possible self-regulation strategies to be used in a particular situation, motivating latent goals that negatively affect well-being (Baumann et al. 2005). Therefore, one can be unconsciously affected by stressors that influence implicit memory and motivation, or metacognition, and initiate specific emotion-regulation strategies that arise from the interplay between explicit and implicit processes (Gyurak et al. 2011).

From this perspective, perseverative thinking becomes relevant for general health and mortality (Brosschot 2002; Verkuil et al. 2010, 2012; Ottaviani et al. 2013). Importantly, health problems arise not only from the tendency to undertake health risk behaviours in people who use perseverative thinking as a regulatory strategy (Clancy et al. 2016), but also from the chronic activation of prolonged stress responses (Padgett and Glaser 2003; Kemp and Quintana 2013; Manenschijn et al. 2013). Perseverative cognition has been shown to impact various physiological systems, including the hemodynamic profile (LeMoult et al. 2016; Brosschot et al. 2017; Cropley et al. 2017; Ottaviani et al. 2017), the immune functioning (Segerstrom et al. 2008; Zoccola et al. 2014; Moriarity et al. 2020), and the endocrine system (Mantella et al. 2008; Zoccola and Dickerson 2012; Cropley et al. 2015; Ottaviani et al. 2016). Generally, perseverative thinking exacerbates health consequences by prolonging cognitive and physiological activation associated with stress, both prior to and following exposure to stressors. This can lead to a cascade of negative physiological responses, such as decreased heart rate variability, and impaired respiratory sinus arrhythmia. Moreover, it increases heart rate, blood pressure, levels of C-reactive protein, pro-inflammatory cytokines, and cortisol.

**Figure 3.**
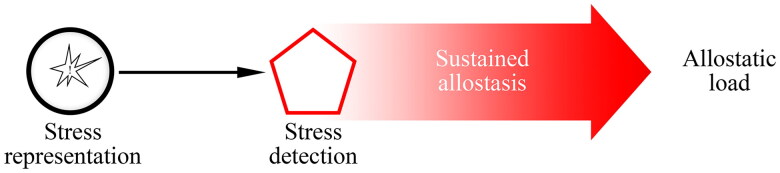
The represented stress-related information acts as a signal to prepare the body for facing actual stressors. If this process is continuously carried out in the long run it will raise the set point for homeostasis or allostatic load.

In summary, perseverative thinking may prolong stress response through the hyperactivation of the allostatic system. In turn, stress may increase the use of maladaptive self-regulation strategies which eventually prolong the stress response. These bidirectional influences can vastly lay below the threshold of awareness (Figure 3).

## Memory updating

As we discussed in the first paragraph, autobiographical memory drives our behaviour. However, we do not remember events only because they happened to us, but also because we further mentally elaborate and evaluate them (Lee 2008, 2009). Cognitive reappraisal is a mechanism defined as the attempt to reinterpret an emotion-eliciting situation in a way that alters its meaning and changes its emotional impact (Gross and John 2003). This strategy can be adaptive or maladaptive, depending on the context (Troy et al. 2013). A series of studies conducted in the last two decades indicate that, during the re-elaboration of memories, our memory traces enter a dynamic state in which it is possible to change their characteristics, a process called memory reconsolidation (Walker et al. 2003; Nader and Einarsson 2010). Importantly, memory retrieval is not sufficient to initiate reconsolidation, as traces must also become ‘labile’ or ‘destabilized’ and thereby susceptible to modification (Tronson and Taylor 2007; Finnie and Nader 2012).

One way in which memory reconsolidation is triggered follows supervised learning models, which posit that the brain adjusts representations to predict outcomes based on perceptual feedback or error correction mechanisms (Rumelhart et al. 1986; Gluck and Myers 1993), involving a mismatch between expected and current events (Sevenster et al., 2014; Exton-McGuinness et al. 2015; Fernández et al. 2016). This mechanism allows us to update our model of the world and our predictions in the light of new information (Courville et al. 2006; Friston 2010; Clark 2013; Dunsmoor et al. 2015). In these processes, learning occurs through the detection of discrepancies between expected and actual outcomes, a process that refines internal representations. Consistent with these views, studies have found that two stimuli are integrated when they predict the same outcome and differentiated when they predict different outcomes (Tompary and Davachi 2017). It has been proposed (Fernández et al. 2017) that when an incomplete reminder ­triggers perseverative thinking, the initial mismatch between the expected and the current event is ­reinterpreted as being congruent with the schema, and consequently the engram reconsolidation occurs in agreement with higher-level negative beliefs. From this perspective, an incomplete reminder can automatically trigger perseverative thinking, which provides new information for an update of the inner model. However, this information is created through the same process over and over again in a maladaptive cycle, reinforcing the model through reconsolidation, every time in the same way. In fact, ineffective defensive strategies aimed at diminishing anxiety responses, like worry, impair the flow of error signals, causing a decrease in the precision of new evidence, and an increase in the weight of the prior beliefs (Fernández et al. 2017).

A second mechanism by which memory reconsolidation occurs follows unsupervised learning rules that adjust neural representations based on the strength of memory activation during the retrieval process (Sinclair and Barense 2019). Unlike supervised learning, which relies on explicit feedback to refine predictions, unsupervised learning models operate by detecting statistical regularities and strengthening co-occurring representations, without direct external reinforcement (Ritvo et al. 2019). These models focus on local synaptic changes driven by internal patterns of activation, rather than explicit consideration of predictive accuracy for external outcomes. A classic example of such a model is the Hebbian rule ‘Neurons that fire together, wire together’ (Sanger 1989). When two memories are simultaneously activated, their connection is strengthened, leading to reduced competition during subsequent retrieval attempts (Ritvo et al. 2019). In this perspective, memory associations are reinforced by the robust concurrent representations of the reminder and context, rather than the reminder’s ability to predict the occurrence of that context (Alberini and LeDoux 2013). Accordingly, it was shown that desire thinking, a cognitive process prefiguring positive addiction-related experiences, can lead to the strengthening of specific associations between neutral cues and the addiction context (Mattioni et al. 2023).

**Figure 4.**
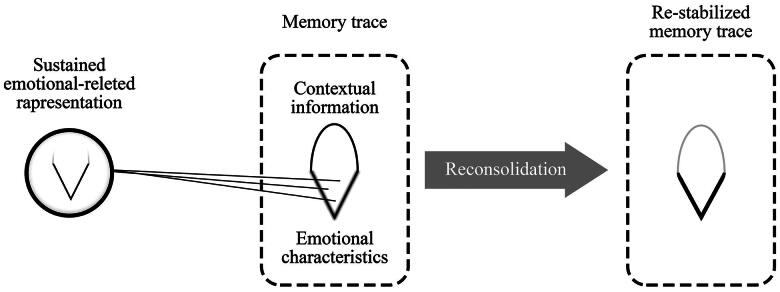
The continuous representation of emotional/motivational characteristics of a situation involves the activation of the related memory trace. This sustained activation may open a reconsolidation window that ends up reinforcing only these characteristics instead of other contextual information.

Since perseverative thinking is focused on emotional characteristics of memories or previsions, constant activation of memory traces might promote their transformation in a labile state and their further re-stabilisation in a way that emphasises the emotional and motivational characteristics. The outcome of this process is the change in the stress response and the emotional state resulting from the subsequent exposure to salient situations (Figure 4). So, for example, the process of worrying before social situations will increase anxiety and avoidant behaviour because the emotional-motivational features of the engrams representing ‘social situation’ will be inflated, at the expense of its contextual features.

## A new model of self-regulation and perseverative thinking

So far, we have seen how perseverative thinking involves multiple subprocesses: detection of the emotional-motivational valence of inputs; selection of a determinate cognitive operation; representation of stress-related information; and implementation of operative processes over this information. The representation of constant stressors that occurs through the perseverative thinking processes only momentarily reduces the stress response. At the same time, these processes activate the allostatic system, prolonging the stress response for the entire organism and strengthening stress-related memories. Lastly, perseverative thinking reinforces metacognitive schemas which favour the selection of these regulation strategies.

**Figure 5.**
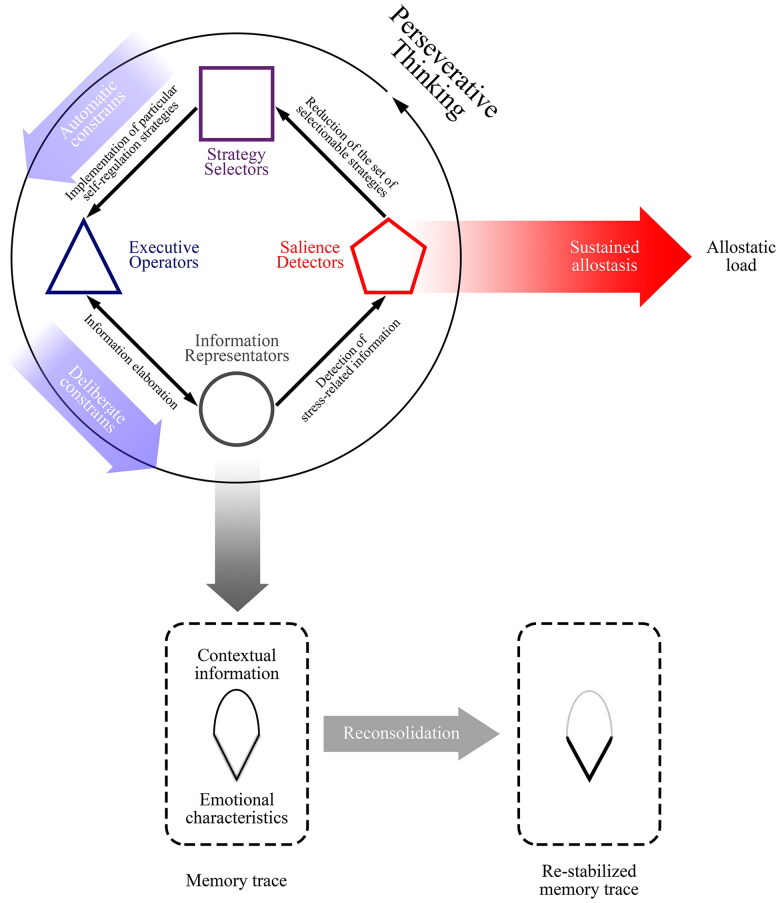
A model for dysfunctional cognitive self-regulation. Perseverative thinking arises when the constraints that these agencies impose on each other become inflexible: information representators constantly broadcast stress-related information, to whom salience detectors respond by reducing potential variability of strategy selectors activity, which end up choosing always to implement the same strategy through the executive operators, which involves making information representators constantly broadcasting stress-related information, and so on.

In this section, we aim to describe a conceptual model of cognitive self-regulation that fits all the aforementioned characteristics and can be applied in different contexts. The proposed model assumes that perseverative thinking comprises four distinct groups of information processing agencies, i.e., interacting parts, each performing a specialised function, that together create complex behaviours (Minsky 1988), each with an independent effect (Figure 5):

- salience detectors, representing the sparse set of discrepancy monitoring systems involved in stress detection;
- strategy selectors, which choose which strategy to implement by constraining the activity of the executive operators;
- executive operators, which represent executive functions for the implementation of self-regulation strategies;
- information representators, which generate and maintain representations decoupled from online perception and broadcast this offline information to other agencies.

In this context, each agency is not a single entity but a group of smaller, function-specific units that work together to manage specific tasks. These four agencies are hierarchically organised, involve conceptually different categories of functions, and may impose constraints on the activity of other agencies to achieve their purpose. Notably, unlike prior models that focus on isolated mechanisms, the key innovation of our framework lies in the description of how different agencies interact to promote and perpetuate perseverative thinking. These agencies do not follow a fixed temporal sequence but operate in parallel, engaging in bidirectional exchanges that shape the overall process. While previous frameworks often suggest a unidirectional progression, our approach underscores the emergence of non-linear temporal dependencies, driven by mutual constraints. This dynamic structure allows for greater flexibility in capturing the complexity of perseverative thinking, moving beyond rigid, hierarchical or sequential models.

At the neural level (BOX 2), each agency should be considered as a population of neurons with similar functions at the mesoscale level, as in the classical pandemonium architecture (Selfridge 1958). In this model, each agency is responsible for recognising certain patterns or features and signalling this recognition to higher-level agencies, which, in turn, integrate the information to form more complex perceptions and decisions. Through the personal history of individuals, memory-based associations between different kinds of agencies are created, and this results in a faster and more automatic selection of particular chains of connection over the years.

Salience detectors are involved in the detection of salient features of stimuli coming from internal and external information processing streams, modulating allostasis accordingly. This function is carried out through the activation of memory-based associations between contextual and emotional-motivational features of a given stimulus, be it conscious or implicit. Each detector is involved in a different stage of information processing: some deal with immediate input characteristics, others with more complex aspects of information. Salience detectors carry out three main functions. First, they can impose different levels of constraints on the strategy selectors, along a continuum that goes from free exploration, e.g. during mind wandering, to less flexible activities when the stress level increases, as in perseverative thinking. Eventually, they can even end up allowing only fight-flight-freeze responses in extreme cases, such as in life-threatening situations or during panic attacks. Second, they are involved in the reconsolidation of motivational/emotional memory when sustainably activated by the information representators. Third, they influence hemodynamic, immune, and endocrine systems depending on the level of the discrepancy, for example by activating allostatic load systems in preparation for a possible stressor or an immediate menace.

Strategy selectors are involved in the selection of strategies, implemented by executive operators, to manage the activity of the salience detectors. Some strategy selectors choose among immediate strategies, others manage short-term strategies, and still others are involved in the maintenance of long-term objectives. Their activity may represent the true procedural part of metacognition. The main function of strategy selectors is to put constraints on the possible actions performed by executive operators in order to implement a particular self-regulation strategy. The selection is carried out by activating the memory-based association between the activity of salience detectors and its resulting effect on executive operators. In this view, inflexible metacognitive schemas are represented by having only a few operational choices, despite the level of variability of salience detectors activity, e.g. allowing the executive operators to perform only the set of processes involved in perseverative thinking.

Executive operators represent various aspects of executive functions involved in different levels of self-regulation activity. Some executive operators perform basic processing activities, others arrange these activities to implement more complex functions, and still others manage the activity of the last ones to accomplish even more strategic goals. Therefore, these activities constitute a nested structure of well-learned processes aimed at implementing self-regulatory objectives. To do so, executive operators use information representators to broadcast different features of stressor-related information, making more data available for additional elaboration. In the case of perseverative thinking, executive operators act on stressor representations in different ways, e.g. depressive rumination passively involves the lack of inhibition for previous mental sets, whereas angry rumination more actively involves difficulties in task switching. Despite these differences, the underlying mechanism is always stereotypical, as it implies continuous operations on angry- and depressive-related information.

Information representators are involved in building internal representations decoupled from online perception, through the integration of memory traces, in order to broadcast internally generated content, independent from immediate sensory input, to the other agencies. Different information representators deal with different functions, from the bottom-up representation of diverse specific features of information to top-down integration into a coherent whole. They automatically activate various levels of memory traces connected to those that are being represented, making new information available. Information representators activity lies on two continuums: intensity and variability. The first varies from very low activity, as when undertaking external tasks that require perceptual attention, to very high activity, such as when one is completely involved in thought. The second varies from very light constraints, when information representators freely integrate memory traces into a representation with great variability, as during mind wandering, to very tight constraints, when the operations regard mainly the same representation over and over, as in perseverative thinking. This sustained reactivation and integration of memory traces may bring them into a labile state in which they can be reconsolidated. The main function of information representators is to broadcast information at various levels to support agency-specific operations. Executive operators strategically manage their intensity and variability to implement functioning, strategy selectors automatically detect emotional-motivational features of the information represented. Importantly, the activity of information representators can also be carried out below the threshold of awareness, explaining implicit perseverative thinking.

The vicious cycle that leads to perseverative thinking can be described as follows: when salience detectors encounter external or internal inputs, they operate on strategy selectors to choose the appropriate self-regulation strategy. Then, strategy selectors make executive operators implement the strategies using the information provided offline by information representators. If the process ultimately succeeds in decreasing the activity of salience detectors, then it will be more probably repeated.

Our model explains how perseverative thinking may affect both perceptual and allostatic systems. The constant activity between information representators and executive operators during perseverative thinking abnormally increases the strength and the emotional motivational features of memory engrams through memory reconsolidation. Furthermore, the activity between information representators and salience detectors influences autonomic activity by continuously sending visceromotor prediction signals to control the internal milieu in order to sustain physiological self-regulation predictions.

Notably, perseverative thinking may arise from different parts of this feedback loop. Thus, our model represents an important advantage in mental disorders description because it allows to look at the global effect of perseverative thinking but, at the same time, also to cluster this process into the constituent agencies which can be studied independently. For example, perseverative thinking can emerge from the hypersensitivity of salience detectors, which may treat too many stimuli as requiring regulation. It can also be caused by inflexible strategy selectors activity, which keeps choosing the same strategy. Similarly, if executive operators always implement the same pattern of actions, the information will be treated in the same way, making cognitive self-regulation redundant and continuously forcing information representators to activate the same stress-related content. Lastly, information representators may automatically integrate memory traces linked with stress-related engrams, constantly activating salience detectors and triggering continuous allostatic preparation.

This framework provides a valuable lens through which to understand how various therapeutic approaches target specific aspects of this complex phenomenon. Behavioural therapies, such as exposure (Powers and Emmelkamp 2008; McGuire et al. 2014), aim to diminish the salience of triggering stimuli by reducing their associated physiological arousal. Techniques like relaxation during imagery exercises and exposure to feared situations seek to counterconditioning these responses. Cognitive-behavioural therapies, recognising that mental disorders arise from biased information processing (Ellis 2010; Beck 2020), prioritise correcting these biases. Within our model, this translates to influencing executive operators, enhancing the flexibility and self-regulation of how information is represented. Cognitive therapies, such as metacognitive therapy (Wells 2011) and schema therapy (Young et al. 2006), empower individuals to recognise and modify maladaptive beliefs and schemas related to perseverative thinking. This process influences strategy selectors, expanding the range and effectiveness of available cognitive strategies. Finally, mindfulness-based stress reduction (Kabat-Zinn 2003; Grossman et al. 2004), cultivates present-moment awareness, shifting the activation of stress-related memories from habitual and often unconscious patterns towards a more conscious and mindful engagement. This fosters more flexible and varied patterns of information representation, effectively opposing the rigid and repetitive patterns characteristic of perseverative thinking. This novel model of self-regulation offers a significant advancement in our understanding of perseverative thinking, providing a robust framework for future research and clinical applications.

**Box 2.** Neurocognitive systems underlying perseverative thinking.

A recent meta-analysis of neuroimaging studies (Makovac et al. 2020) has shown that perseverative thinking engages a large set of brain regions that partially overlap with key nodes of the default mode network (DMN), i.e. the medial prefrontal cortex (PFC), the posterior cingulate cortex, the inferior parietal lobule, and the medial and lateral temporal cortices (Di and Biswal 2014; Raichle 2015). In this view, the interaction between prefrontal and cingulate regions with insular areas may support the characteristic conjunction of self-referential and affective processing with cognitive control and autonomic arousal (Makovac et al. 2020). These findings are supported by high-confidence evidence due to the convergence of multiple studies and meta-analyses. While variations in task paradigms (e.g. worry vs. rumination induction) introduce some variability, they do not substantially affect the interpretation of the underlying network dynamics. Healthy controls and clinical groups undertaking perseverative thinking can also be distinguished based on the differential activity of the temporal gyrus (Makovac et al. 2020), which is involved in working memory maintenance (Park et al. 2011), speech representation (Chang et al. 2010), semantic memory (Martin and Chao 2001), and conceptual processing (Wei et al. 2012). Additionally, differences are observed in the activity of the occipital gyrus (Makovac et al. 2020), which is involved in the unconscious selection of predictable information (Tu et al. 2013) and mood-congruent processing that biases attention to negative emotional information (Teng et al. 2018).

**Figure 6.**
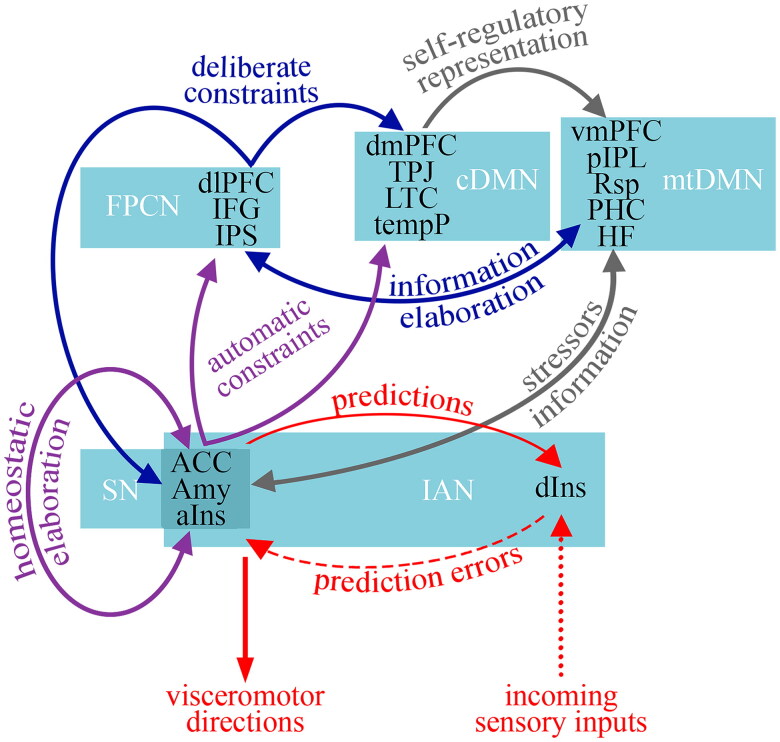
The proposed neural model which sustains perseverative thinking. The Interoception/Allostasis Network (IAN) detects salient information for self-regulation and modulates allostasis accordingly. Accordingly, the Salience Network (SN) exercises constraints on the Frontoparietal Control Network (FPCN) and the core default mode network (cDMN) activity in order to maintain cognitive self-regulation on a reduced set of contents. As a consequence, the medial temporal DMN (mtDMN) may redundantly represent stressors-related information, which ends up activating continuously the IAN. Red arrows represent salience detectors activity; purple arrows represent strategy selectors activity; blue arrows represent executive operators activity; grey arrows represent information representators activity. dlPFC: dorsolateral prefrontal cortex; IFG: inferior frontal gyrus; IPS: intraparietal sulcus; dmPFC: dorsomedial prefrontal cortex; TPJ: temporoparietal junction; LTC: lateral temporal cortex; tempP: temporal pole; vmPFC: ventromedial prefrontal cortex; pIPL: posterior inferior parietal lobe; Rsp: retrosplenial cortex; PHC: parahippocampal cortex; HF: hippocampal formation; ACC: anterior cingulate cortex; Amy: amygdala; aIns: anterior insula; dIns: dorsal insula.

The neural networks hypothesised to sustain the theoretical agencies are well-characterised in neuroimaging literature, but direct causal evidence linking these networks to perseverative thinking is still lacking. The following sections isolate the neural components involved in perseverative thinking based on the brain networks that are supposed to sustain the various agencies and their interactions, according to the current understanding of the brain functional architecture (Figure 6).

### Salience detectors

Salience detectors are involved in the detection of salient features of stimuli coming from internal and external information processing streams and modulating allostasis accordingly. Barrett and colleagues (2016) propose that perseverative thinking may be a behavioural hallmark of inefficient allostasis: by providing biased prediction errors, perseverative thinking issues perceptual predictions that remain uncorrected by sensory feedback (Fernández et al. 2017). From a neural perspective, the representation of the ‘world’ includes not only sensations from the external environment but also interoceptive sensations from the internal milieu of the body, which colour the conscious experience of feelings and emotions in order to maintain homeostasis (Barrett and Simmons 2015). Allostasis and interoception are maintained within an integrated system (Kleckner et al. 2017) involving the ACC, the ventral anterior insula, and the amygdala that initiates visceromotor directions to the hypothalamus and brainstem nuclei to regulate the autonomic, neuroendocrine, and immune systems. These visceromotor control regions also send anticipated sensory consequences of visceromotor changes to the primary interoceptive cortex in the dorsal sector of the mid to posterior insula, which receives the incoming sensory inputs from the internal milieu of the body through the vagus nerve. Variations in task design, ranging from passive interoception to active regulation, introduce moderate confidence in how these regions contribute to perseverative thinking. However, we have high confidence in their role in salience detection and allostasis due to consistent activation patterns in the ACC and insula across different studies. Furthermore, the ventral subnetwork of the salience network, which involves connections between the ventral anterior insula and ACC (Touroutoglou et al. 2012), appears fundamental for directing selection in the visceromotor system responses that support homeostasis (Barrett and Satpute 2013). Importantly, anticipations about bodily states are updated when discrepancies between expected and actual bodily sensations are relayed to visceromotor regions. These errors act as learning signals, prompting adjustments to future predictions to better align with incoming sensory data.

Thus, a single brain system appears to support allostasis and interoception, influencing various psychological functions such as emotion, pain, memory, and decision-making. The functions of salience detectors largely overlap with the functions attributed to this system, which allows the identification of potential stressors, the appropriate modulation of allostasis, the transfer of information to other brain areas involved in cognitive self-regulation, and the update of interoceptive memory.

### Strategy selectors

Strategy selectors are involved in the selection of self-regulatory strategies, implemented by executive operators, to manage the activity of the salience detectors. At the neural level, one set of brain regions consisting of ACC, amygdala, and anterior insula, i.e. the so-called salience network, has been strongly linked to the perception and the response to homeostatic demands, by receiving from areas involved in enteroception and allostasis and projecting to areas involved in cognitive control (Seeley 2019). It has been proposed (Christoff et al. 2016) that this network can exert automatic constraints on the output of the medial temporal lobe components of the DMN. This can happen in two ways: directly through the core DMN subsystem (Andrews-Hanna et al. 2010) and indirectly through the frontoparietal control network (FPCN; Dixon et al. 2018). The FPCN sustains the activity of executive operators, as shown below. As a result, this interaction limits the variability of thought, which in turn affects the activity of information representators. Furthermore, during perseverative thinking, the activity of the ACC distinguishes patients from healthy controls (Makovac et al. 2020). While we maintain moderate confidence in the ACC’s specific role in perseverative thinking due to variable activation patterns across tasks (e.g. Stroop vs. self-referential tasks), we have high confidence in its broader role given its consistent involvement in cognitive control studies. This region integrates signals coding for the expected payoff from a controlled process, the amount of control that must be invested to achieve that payoff, and the cost in terms of cognitive effort (Botvinick et al. 2004). This information converges to determine whether, where, and how much control to allocate, and to select among candidate control functions, which are then implemented through the involvement of a different brain region, i.e., the lateral PFC (Holroyd and Yeung 2012; Shenhav et al. 2013). In turn, this latter region houses a discrete set of hierarchically organised subregions, underpinning different levels of control functions (Badre 2008; Badre and D’esposito 2009) involved in different aspects of metacognition (Fleming et al. 2012) such as memory control (Depue et al. 2010), monitoring and selection processes (Wagner et al. 2001).

In synthesis, the salience network may sustain strategy selectors activity by receiving information from the parts of the brain involved in salience detectors activity, in order to constrain the implementation of executive function on a reduced set of information.

### Executive operators

Executive operators represent various aspects of executive functions involved in different levels of self-regulation activity. Dedicated PFC networks create a functional architecture for efficient processing without the persistent need for reconfiguring circuits, but flexible enough for reconfiguration to occur when more flexible behaviours are needed based on contextual demands (Menon and D’Esposito 2022). The FPCN contributes to executive control, the ability to deliberately guide action based on goals. This network is primarily composed of the dorsolateral PFC and posterior parietal regions located in between the lateral parietal components of the dorsal attention network, i.e. a set of brain regions, that are putatively involved in attentional control based on goals or expectations (Sestieri et al. 2017) and DMN, such as inferior frontal gyrus (Hampshire et al. 2010) and intraparietal sulcus (Marek and Dosenbach 2018). It can be divided into two distinct subsystems (Dixon et al. 2018). The first subsystem is closely connected to the dorsal attention network and plays a key role in regulating visuospatial attention, ensuring that focus remains on task-relevant perceptual information rather than task-irrelevant thoughts (Yin et al. 2022). The second subsystem exhibits stronger connectivity with the DMN and contributes to executive control in introspective processes (Dixon et al. 2018; Yin et al. 2022). This latter subsystem enables modes of thought that are relatively free from the constraints of concrete sensorimotor interactions with the environment. The FPCN can exert deliberate constraints on thought by flexibly coupling with the core DMN and the salience network, thus momentarily reinforcing or reducing the automatic constraints for maintaining goal-directed activity (Christoff et al. 2016). In this view, the FPCN may flexibly couple with the default and dorsal attention networks according to the task domain, serving as a cortical mediator linking the two networks in support of goal-directed cognitive processes (Spreng et al. 2010). Indeed, task-related functional connectivity analyses demonstrate that the default network can be involved in goal-directed cognition when its activity is coupled with the FPCN (Spreng et al. 2010). Even if this finding is based on a relatively small sample size (*n* = 20) these executive control mechanisms are widely accepted. Discrete executive functions are supported by this superordinate network (Niendam et al. 2012), which are intrinsically linked with self-regulation, supporting important mechanisms in individual self-regulatory goal pursuits and regulating distress (Hofmann et al. 2012).

Executive operators may represent just this activity of implementing executive functions to operate on the information broadcasted from the DMN in order to maintain self-regulatory goals, while the potential variability of this information is limited by brain systems concerning strategy selectors.

### Information representators

Information representators are involved in building internal and perceptually decoupled representations, through the integration of memory traces, in order to broadcast offline information to other agencies. At the neurobiological level, internally directed thinking has been closely associated with the activity of the DMN (Mason et al. 2007). Neural activity within regions of the DMN may reflect abstract features of cognition, which do not directly form the basis of experiences, but instead convey higher-order information about their characteristics (Smallwood et al. 2021). There is high confidence in the DMN’s involvement in abstract cognitive processes and internally directed thought, as supported by robust meta-analytic evidence (Mason et al. 2007; Buckner et al. 2008). However, the DMN’s precise causal role in perseverative thinking remains theoretical. Spontaneous thought also recruits non-DMN regions, including the rostrolateral PFC, dorsal anterior cingulate cortex, insula, temporopolar cortex, secondary somatosensory cortex, and lingual gyrus (Fox et al. 2015). The additional involvement of these regions may reflect the activation of executive functions and interoception-related activity linked to spontaneous thought, which we mentioned before. In particular, the medial temporal component of the DMN seems to act as a representational hub that integrates information from memory while the core component of the DMN deals with self-regulatory decisions (Andrews-Hanna et al. 2010). The DMN seems also to be involved in high-level prediction-error representations (Brandman et al. 2021) and consequently in memory updating (Pine et al. 2018). Distinct memories are associated with different memory traces in the hippocampus. In this respect, in the murine model (Cardin et al. 2010), the artificial activation of positive, neutral, or negative engrams across the longitudinal axis of the hippocampus differentially modulates behavioural outputs (Chen et al. 2019). Chronic reactivation of dorsal hippocampal cells, which encode spatial, temporal, and contextual information (Jung et al. 1994; Moser et al. 1995), results in a long-lasting context-specific reduction in freezing and place preference. In contrast, chronic reactivation of ventral hippocampus cells, which are involved in stress responses and emotional state (Herman et al. 2005; Xu et al. 2016; Parfitt et al. 2017; Jimenez et al. 2018), results in a durable context-specific enhancement of freezing and place preference (Chen et al. 2019).

Information representators are expected to function in the same way, broadcasting general information to the other agencies, which represents the coupling of DMN and other systems, and reconsolidating differentially contextual and emotional/motivational memories by sustainably representing that information.

## Conclusions

The present review focused on perseverative thinking, a maladaptive self-regulation strategy that maintains stress-related information and causes continuous allostatic preparation, which wears and tears the body. We have described how this maladaptive process is maintained through modifications of the procedural and representational memories, increasing the probability that situations are misinterpreted as requiring regulation, and thereby triggering even more perseverative thinking. We also discussed how, in the last decades, perseverative thinking has received ever-increasing importance for the conceptualisation of mental and somatic health diseases, as testified by the wide variety of related constructs that have been presented in the literature.

The model we propose is not an alternative to previous frameworks of perseverative thinking but rather represents an integration of their strongest points. However, there are some differences between the current perspective and traditional theories. Firstly, our model applies to the various forms of perseverative thinking, showing how they influence memory reconsolidation and autonomic anticipation. Secondly, it is not confined to a specific field of investigation, as it applies to neurobiological, cognitive, and therapeutic paradigms. In particular, the agencies can be easily seen in terms of either cognitive or neural entities and their integration explains the influence of perseverative thinking on memory and somatic health, handling the complexity and variability of cognitive self-regulation. Third, the model considers different sources of perseverative thinking along with their respective treatment, explaining why even different interventions can have positive effects. In short, our model allows us to focus on particular aspects of perseverative thinking, opening new possibilities for clinical and scientific processes.

A substantive amount of work has been carried out to date which can enable researchers to build a global theory of psychopathology. In this review, we have shown how perseverative thinking may account for many aspects of mental and somatic diseases, and how it could be an important addition to the existing models of illness. Much remains to be done to construct an all-inclusive framework for mental disorders. Part of this task will likely involve the integrated work of researchers coming from different fields and perspectives.
